# Quantification and Monitoring of the Effect of Botulinum Toxin A on Paretic Calf Muscles of Children With Cerebral Palsy With MRI: A Preliminary Study

**DOI:** 10.3389/fneur.2021.630435

**Published:** 2021-04-16

**Authors:** Claudia Weidensteiner, Philipp Madoerin, Xeni Deligianni, Tanja Haas, Oliver Bieri, Tugba Akinci D'Antonoli, Katrin Bracht-Schweizer, Jacqueline Romkes, Enrico De Pieri, Francesco Santini, Erich Rutz, Reinald Brunner, Meritxell Garcia

**Affiliations:** ^1^Division of Radiological Physics, Department of Radiology, University Hospital of Basel, Basel, Switzerland; ^2^Department of Biomedical Engineering, University of Basel, Allschwil, Switzerland; ^3^Department of Radiology, University Hospital of Basel, Basel, Switzerland; ^4^Department of Radiology, University Children's Hospital Basel, Basel, Switzerland; ^5^Laboratory for Movement Analysis, University Children's Hospital Basel, Basel, Switzerland; ^6^Murdoch Children's Research Insitute, The University of Melbourne, Pediatric Orthopedic Department, The Royal Children's Hospital, Parkville, VIC, Australia; ^7^Faculty of Medicine, The University of Basel, Basel, Switzerland; ^8^Department of Orthopedic Surgery, University Children's Hospital Basel, Basel, Switzerland; ^9^Department of Radiology, Division of Neuroradiology, University Hospital of Basel, Basel, Switzerland

**Keywords:** cerebral palsy, MRI, botulinum toxin A, T_2_, diffusion, fat fraction, calf muscles, pediatric

## Abstract

**Background:** Muscles from patients with cerebral palsy (CP) are often spastic and form contractures that limit the range of motion. Injections of botulinum toxin A (BTX) into the calf muscles are an important treatment for functional equinus; however, improvement in gait function is not always achieved. BTX is also used to test muscle weakening for risk evaluation of muscle lengthening surgery. Our aim was to assess the effect of BTX over time on calf muscle properties in pediatric CP patients with MRI.

**Material and Methods:** Six toe-walking CP patients (mean age 11.6 years) with indication for lengthening surgery were prospectively enrolled and received BTX injections into the gastrocnemius and soleus muscles. MRI scans at 3T of the lower legs and clinical examinations were performed pre-BTX, 6 weeks (6w), and 12 weeks (12w) post-BTX. A fat-suppressed 2D multi-spin-echo sequence was used to acquire T_2_ maps and for segmentation. Fat fraction maps were calculated from 3D multi-echo Dixon images. Diffusion tensor imaging (DTI) with a 2D echo-planar imaging (EPI) sequence yielded maps of the mean apparent diffusion coefficient (ADC) and of the fractional anisotropy (FA). Hyperintense regions of interest (ROIs) on the T_2_-weighted (T_2_w) images at 6w were segmented in treated muscles. Mean values of T_2_, fat fraction, ADC, and FA were calculated in hyperintense ROIs and in reference ROIs in non-treated muscles.

**Results:** Hyperintensity on T_2_w scans and increased T_2_ (group mean ± standard deviation: 35 ± 1 ms pre-BTX, 45 ± 2 ms at 6w, and 44 ± 2 ms at 12w) were observed in all patients at the injection sites. The T_2_ increase was spatially limited to parts of the injected muscles. FA increased (0.30 ± 0.03 pre-BTX, 0.34 ± 0.02 at 6w, and 0.36 ± 0.03 at 12w) while ADC did not change in hyperintense ROIs, indicating a BTX-induced increase in extracellular space and a simultaneous decrease of muscle fiber diameter. Fat fraction showed a trend for increase at 12w. Mean values in reference ROIs remained unchanged.

**Conclusion:** MRI showed limited spatial distribution of the BTX-induced effects in pediatric CP patients. It could be a promising non-invasive tool for future studies to test BTX treatment protocols.

## Introduction

Cerebral palsy (CP) is a sensorimotor dysfunction caused by damage to the developing brain and is the most common cause of lifelong motor disability in children (1), affecting movement and muscle coordination. Muscles from CP patients are often spastic and form contractures that limit the range of motion (RoM) and joint function. Botulinum toxin A (BTX) has been established as an important treatment modality, particularly for the management of spasticity (2) to reduce inadequate muscle activity in CP patients. BTX is injected locally into the affected calf muscles to control functional equinus and spasticity and thus improve gait function. In general, equinus treatment with BTX is moderately effective, especially in younger children under 6 years of age, with the clinical effect lasting for 3–6 months (3). There is no uniform BTX treatment strategy in CP; instead, there are several recommendations and guidelines about total doses, injection volume, and injection sites based on expert opinions and small clinical trials (2). However, it is unknown to what extent BTX is distributed into the muscle tissue and if it affects the muscle belly as a whole or only partially. BTX blocks the neurotransmission at the motor end plates, which are located in a specific zone within the muscle belly (4). Depending on the injection technique and on the subsequent diffusion of the drug, the complete zone of motor end plates or only a part of it is reached and affected by BTX. This leads to a variation in treatment response (4). Also, improvement in gait function is not always achieved with BTX treatment, especially in children over 6 years of age who may need a surgical lengthening of the calf muscles as an effective treatment for equinus (3). Thus, there is a need to study and monitor the effect on injected muscles.

There are various methods based on magnetic resonance imaging (MRI) that offer insight into muscle tissue changes. An increase in water content, i.e., muscle edema, leads to an increase in the MR transverse relaxation time T_2_ value that manifests as hyperintense areas on T_2_-weighted (T_2_w) images. Muscle T_2_w MR images and mainly quantitative T_2_ relaxation values of water can assess inflammation, e.g., in thigh muscles in dermatomyositis (5, 6), and edema, e.g., in calf muscles in Duchenne dystrophy (7). The measurement of fat infiltration and replacement in muscles monitors the extent and severity of muscle destruction, especially in chronic diseases like Duchenne muscular dystrophy (8) or diabetic neuropathy (9). Diffusion-weighted imaging (DWI) and diffusion tensor imaging (DTI) are methods to investigate the tissue microstructural integrity since cell membranes and other structures restrict the diffusion of water molecules. In DTI, a diffusion tensor describing the diffusivity in different directions is generated from a series of diffusion-weighted images. From the eigenvalues of these tensors, the mean apparent diffusion coefficient (ADC; also called mean diffusivity MD in DTI) and the fractional anisotropy (FA)—a measure for diffusion anisotropy ranging from 0 (completely isotropic) to 1 (completely anisotropic)—can be calculated (10). These parameters vary with architectural changes occurring in muscles, e.g., in calf muscle injury (11) or in the lower leg of patients with muscle dystrophy (12).

Our aim was to assess the effect of BTX on muscle properties in pediatric CP patients with MRI over 12 weeks after the injections. We were interested in quantifying the following potential muscle tissue changes using MRI: the T_2_ of muscle water, diffusion of muscle water, and fat content in the BTX-treated muscles. Results from this pilot study may help to better understand the physiological and therapeutic effects of BTX on muscles in patients with CP.

## Materials and Methods

### Participants

Six ambulatory patients diagnosed with CP (five boys and one girl, mean age 11.6 years, range 9.8–12.8 years, three bilateral spastic CP functionally diparetic and three unilateral spastic CP functionally hemiparetic) were included and prospectively monitored between 2018 and 2020. All patients were toe-walking and were scheduled for muscle lengthening surgery with a preoperative BTX test injection in the gastrocnemius muscles to investigate muscle weakening, which can be a side effect of the muscle lengthening surgery (13). Exclusion criteria were previous surgeries on the affected limb(s), claustrophobia, and difficulties in following instructions in the scanner. Full participant characteristics can be found in Table 1. Functional mobility level was classified as Gross Motor Function Classification System level (GMFCS) I (*n* = 4), II (*n* = 1), and III (*n* = 1) (14). Prior to participation, informed written consent was obtained from the participants' parents and additionally from those participants age 12 years or older. The study was approved by the local ethics committee.

**Table 1:** Patient characteristics, treatment, and results of the clinical examination pre-BTX, 6 weeks (6w), and 12 weeks (12w) post-BTX showing lower limb spasticity, passive range of motion (RoM), and manual muscle testing (MMT).

**Patient number**	**Patient characteristics**						**Treatment**				**Spasticity** **(Modified Ashworth scale)**			**Passive RoM** **(deg.)**		**MMT** **(Medical Research Council scale)**	
											**Pre/6w/12w**			**Pre/6w/12w**		**Pre/6w/12w**	
	**Age range pre-BTX (years)**	**Height pre-BTX (cm)**	**Body weight pre-BTX (kg)**	**BMI percentile pre-BTX**	**CP uni/bi**	**GMFCS**	**Side**	**BTX treatment naïve**	**BTX dose**	**BTX dose per body weight (U/kg)**	**PF (at 90^°^ KF)**	**PF (at KE)**	**KF**	**DF (at KE)**	**KE (at HE)**	**PF**	**KF**
1	12–14	146.5	32.7	5	uni	I	r	n	GM, GL, S: 50 U diluted in 1 ml at 1 injection site each	4.6	1+/1+/1	1+/1/1+	0/1/1	−10/−5/−10	0/0/0	2+/2+/2+	5/4/4
2	10–12	151.0	53.8	95	uni	I	r	n	GM, GL, S: 50 U diluted in 1 ml at 1 injection site each	2.8	0/1/1	1/1/0	0/0/1+	−20/−20/−30	−15/−20/−10	2+/2+/2+	4/4/4
3	10–12	146.0	34.4	23	uni	I	r	n	GM, GL, S: 50 U diluted in 1 ml at 1 injection site each	4.4	1+/1/1	1/1/1	0/0/0	15/10/5	0/0/0	2+/2+/3	5/5/5
4	10–12	137.0	32.6	49	bi	I	l	y	GM, GL, S: 50 U diluted in 1 ml at 1 injection site each	4.6	1/0/1	1/0/0	0/0/0	−10/−15/−15	−20/−20/−15	2+/2+/2+	4/4/4
5	10–12	135.0	26.6	3	bi	II	l	n	GM, GL, S: 75 U diluted in 1.5 ml distributed at 2 injection sites each	8.5	1+/2/1	4/1/1+	2/1/1	−30/−15/−15	−5/0/−10	3/2/2+	4/3+/3
6	8–10	127.5	22.7	4	bi	III	l	n	GM, GL: 100 U diluted in 2 ml distributed at 2 injection sites each	8.8	1/1+/1+	1/1+/0	1/1+/1	−5/−15/−15	−10/−15/−10	3+/2+/2+	3/3+/3+

*BMI, body mass index; GMFCS, Gross Motor Function Classification System; GM, m. gastrocnemius medialis; GL, m. gastrocnemius lateralis; S, m. soleus; U, units; BTX, botulinum toxin A; CP, cerebral palsy; uni, unilateral; bi, bilateral; PF, plantarflexor muscles; KF, knee flexor muscles; DF, dorsiflexion; KE, knee extension; HE, hip extension*.

### Treatment

BTX (onabotulinum toxin A, Botox, Allergan plc, Dublin, Ireland) was injected under ultrasound guidance in the gastrocnemius lateralis (GL), medialis (GM), and soleus (S) muscles (dose 50–100 units per muscle, one to two injection sites per muscle, dilution: 50 units diluted to 1 ml). The injection sites were at a horizontal line separating the proximal 1/4 and the distal 3/4 of the muscle belly for the GM and GL and at a horizontal line separating the proximal 1/3 and the distal 2/3 of the muscle belly for the S. The dose was chosen according to the current state of the art recommendations (2, 15): the maximal dose per injection site was 50 units, and the dose per kg body weight was below 20 units/kg (Table 1). Target muscles were determined according to a prior gait analysis. The patients with bilateral paresis were injected in the more severely affected leg only. The injection was not intended to improve the patients' clinical symptoms but to test their reaction on weakness of this muscle for a risk evaluation of the planned lengthening surgery (13).

### Study Protocol

MRI scans and routine clinical examinations were performed pre-BTX injection, as well as 6 weeks (6w) and 12 weeks (12w) post-BTX injection. Gait analysis [as described in Rutz et al. (13)] was performed on the same time points, but it is not reported here because it is out of the scope of this paper. The maximum BTX effect was expected 6w post-BTX (16). The second MRI scan was within 3 months after the first one.

### Clinical Examination

Besides general parameters such as weight, height, and body mass index [BMI (17)], the passive RoM of the knee and ankle joints [passive RoM, using a goniometer (18)] and lower limb spasticity [Modified Ashworth Scale (19)] were measured. Manual muscle testing [MMT; Medical Research Council scale (20)] was performed in the legs to assess muscle strength.

### MR Image Acquisition and Analysis

MRI exams were performed at a whole-body clinical scanner with 3T field strength (Siemens Prisma, Siemens Healthineers, Erlangen, Germany). The patients were positioned feet first supine on the MRI patient table, and the more severely affected leg was restrained with straps at a comfortable resting angle, with the knee in maximal extension. A Siemens 18-element-body array coil was placed on the lower legs. For T_2_ mapping, a 2D multi-spin-echo sequence was used: 32 axial slices, voxel size 1.0 × 1.0 × 3.0 mm^3^, reconstructed matrix size = 320 × 324 × 32, repetition time (TR) = 4.3 s, echo time (TE) = 11–115 ms, fat-saturated, acceleration factor 2, acquisition time 2 min 6 s (21). A 3D multi-echo Dixon protocol was employed to acquire fat-only and water-only images to calculate the fat fraction: VIBE sequence; 6 echoes; voxel size 1.1 × 1.1 × 3.0 mm^3^; reconstructed matrix = 320 × 190 × 96; TR = 20 ms; TE = 1.41, 2.87, 4.33, 5.79, 7.25, and 8.71 ms; flip angle = 12°; acceleration factor 2; and acquisition time 4 min 49 s (22). DTI with a 2D echo-planar imaging (EPI) sequence was used to acquire maps of the ADC and the FA: 46 consecutive sagittal slices, voxel size 2.5 × 2.5 × 2.5 mm^3^, reconstructed matrix = 128 × 52 × 46, TR = 4.0 s, TE = 49 ms, monopolar diffusion gradients in 30 directions with b-values of b = 0 and 700 s/m^2^, fat-saturated, partial Fourier acquisition factor 7/8, acquisition time 2 min 18 s. Maps of T_2_, ADC, FA, and fat fraction (defined as the signal intensity of the fat-only images divided by the sum of the signal intensities of fat-only and water-only images) were calculated online by the scanner software.

Image analysis was performed in the BTX-treated legs only. For each patient, segmentation of hyperintense regions of interest (ROIs) on T_2_w images (TE = 34 ms, from the multi-spin-echo acquisition) of the second MRI time point (6w post-BTX) was performed by an experienced medical imaging technologist (PM) with ITK SNAP 3.6 using thresholding, region growing, and manual correction. A reference ROI (volume 1–2 cm^3^) was drawn manually in the anterior muscle compartment (not BTX treated) with ITK snap. MR images were co-registered with FSL 5.0.11. First, within the same time point, the T_2_w image (TE = 34 ms) and the Dixon acquisition image (TE = 2.87 ms) were co-registered on the b0 image (=image without diffusion weighting) of the DTI dataset. Second, between the time points, T_2_w images pre-BTX and 12w post-BTX were co-registered on the T_2_w image 6w post-BTX. The calculated transformation matrices were used to transfer both the hyperintense and reference ROIs onto the T_2_ maps, ADC maps, FA maps, and fat fraction maps of each time point. The ROI mean values were calculated for each map.

The whole muscle delineation was done on the Dixon acquisition images (TE = 2.87 ms) at 6w time point by an experienced radiologist (TAD) using an in-house developed segmentation tool. Then the volumes of the GM, GL, and S in the treated legs were calculated. For calculating the relative ROI volume, the volume of the hyperintense ROI (after transfer on the Dixon acquisition images) was divided by the summed-up volume of the target muscles (GM+GL+S for patients 1 to 5; GM+GL for patient 6).

## Results

### Clinical Examination

Clinical examinations did not show any relevant changes after treatment in five of six patients: there were no changes larger than 1 in the scores for spasticity and muscle strength (MMT) and no changes over 10° for RoM between pre-injection and 6w or 12w post-injection (Table 1). Only patient 5 showed an improvement in two out of six parameters (passive RoM for dorsiflexion and spasticity for the plantarflexor muscles).

### Magnetic Resonance Imaging

**Figure 1:**
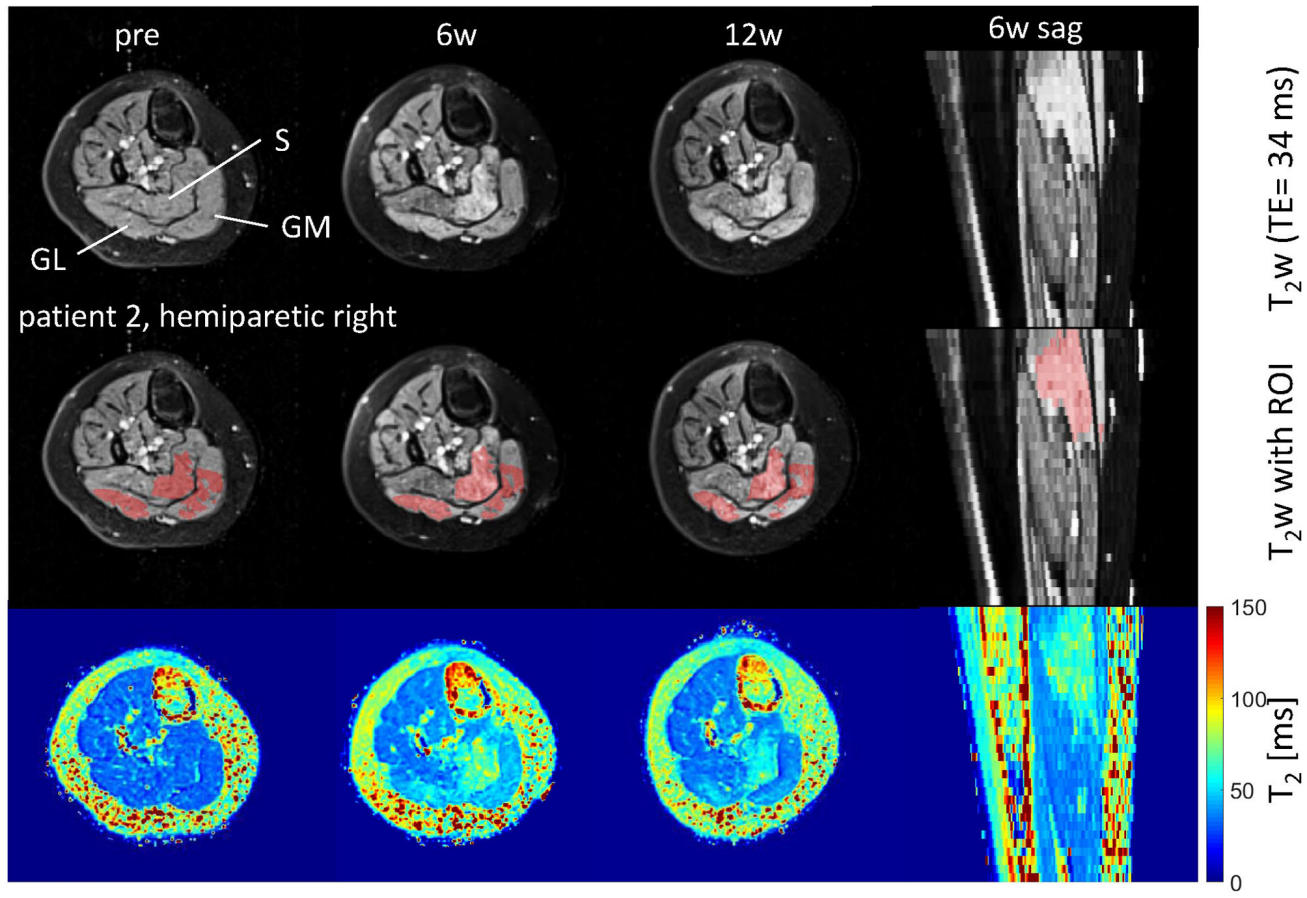
Axial and sagittal T_2_-weighted images in the calf of patient 2 (hemiparetic) pre-BTX and 6 weeks (6w) and 12 weeks (12w) post-BTX (top row) showing hyperintensity at the sites of injection in the soleus (S), gastrocnemius medialis, and lateralis (GM, GL). A region of interest (ROI) comprising the hyperintense regions was segmented and is shown as red overlay in the middle row. The increased T_2_ post-BTX can be seen in the calculated T_2_ maps (bottom row). BTX, botulinum toxin A; y, years; T_2_w, T_2_-weighted; TE, echo time; sag, sagittal.

**Figure 2:**
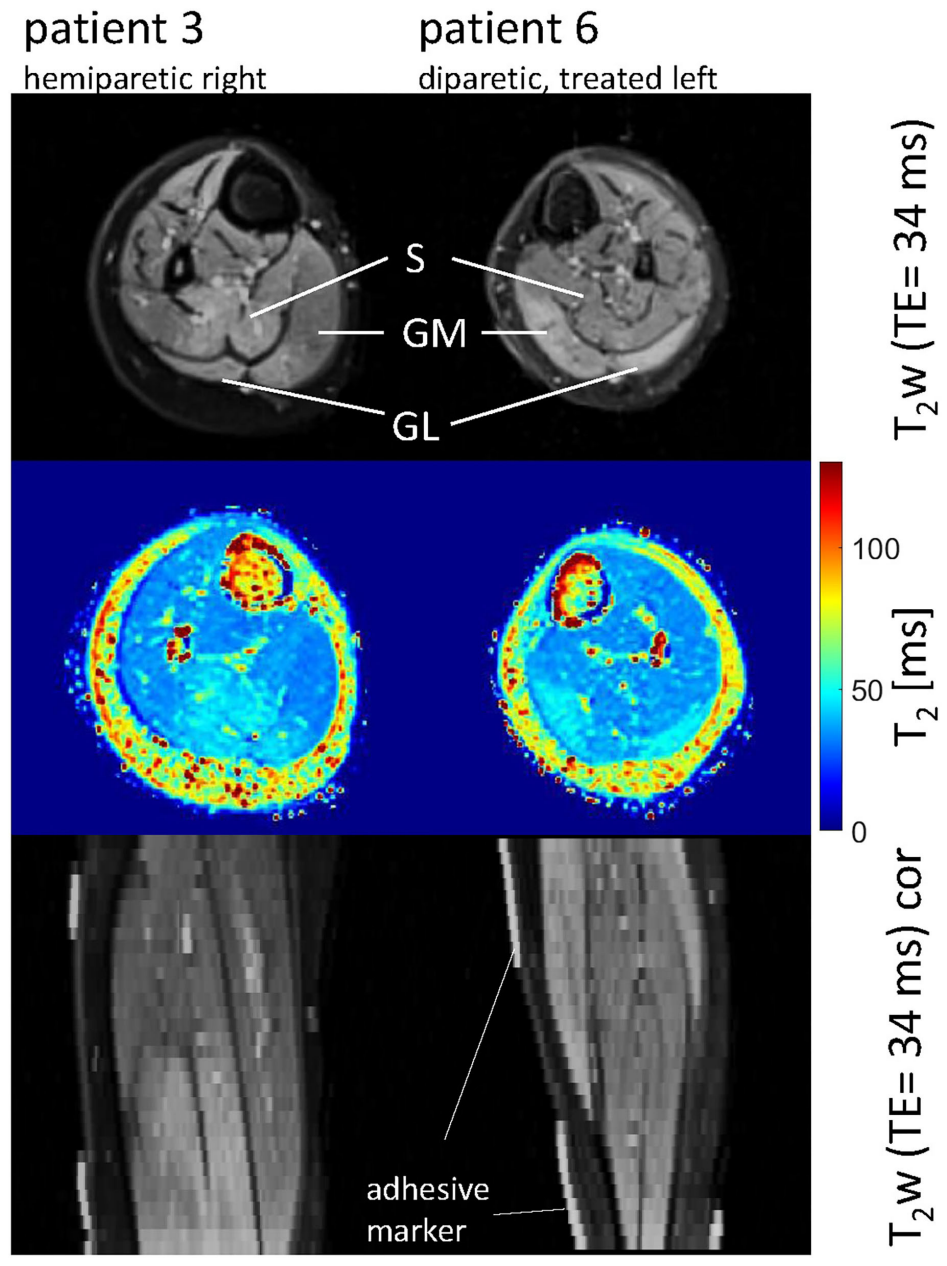
Axial and coronal T_2_-weighted images in the calves of patient 3 (hemiparetic) and patient 6 (diparetic) 6 weeks post-BTX (top and bottom row) showing hyperintensity at the sites of injection in the soleus (S), gastrocnemius medialis, and lateralis (GM, GL) for patient 3, and the GM and GL for patient 6. The increased T_2_ post-BTX can be seen in the calculated T_2_ maps (middle row). BTX, botulinum toxin A; y, years; T_2_w, T_2_-weighted; TE, echo time; cor, coronal.

**Figure 3:**
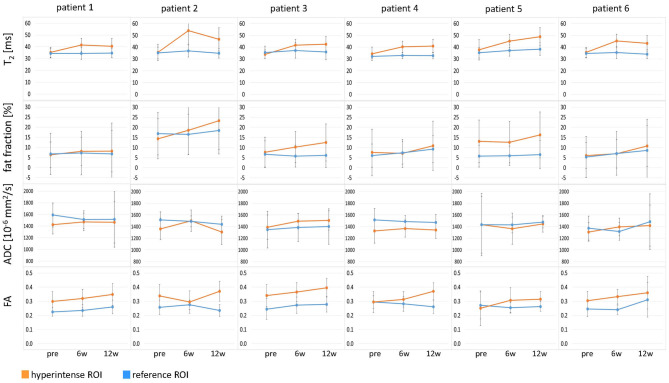
Time courses of T_2_, fat fraction, apparent diffusion coefficient (ADC), and fractional anisotropy (FA) in all six patients at time points pre-BTX, 6 weeks (6w), and 12 weeks (12w) post-BTX. The values are displayed as mean values in the hyperintense ROI (orange) and the reference ROI (blue). The error bars show the standard deviation in the ROIs. ROI, region of interest; BTX, botulinum toxin A.

**Figure 4:**
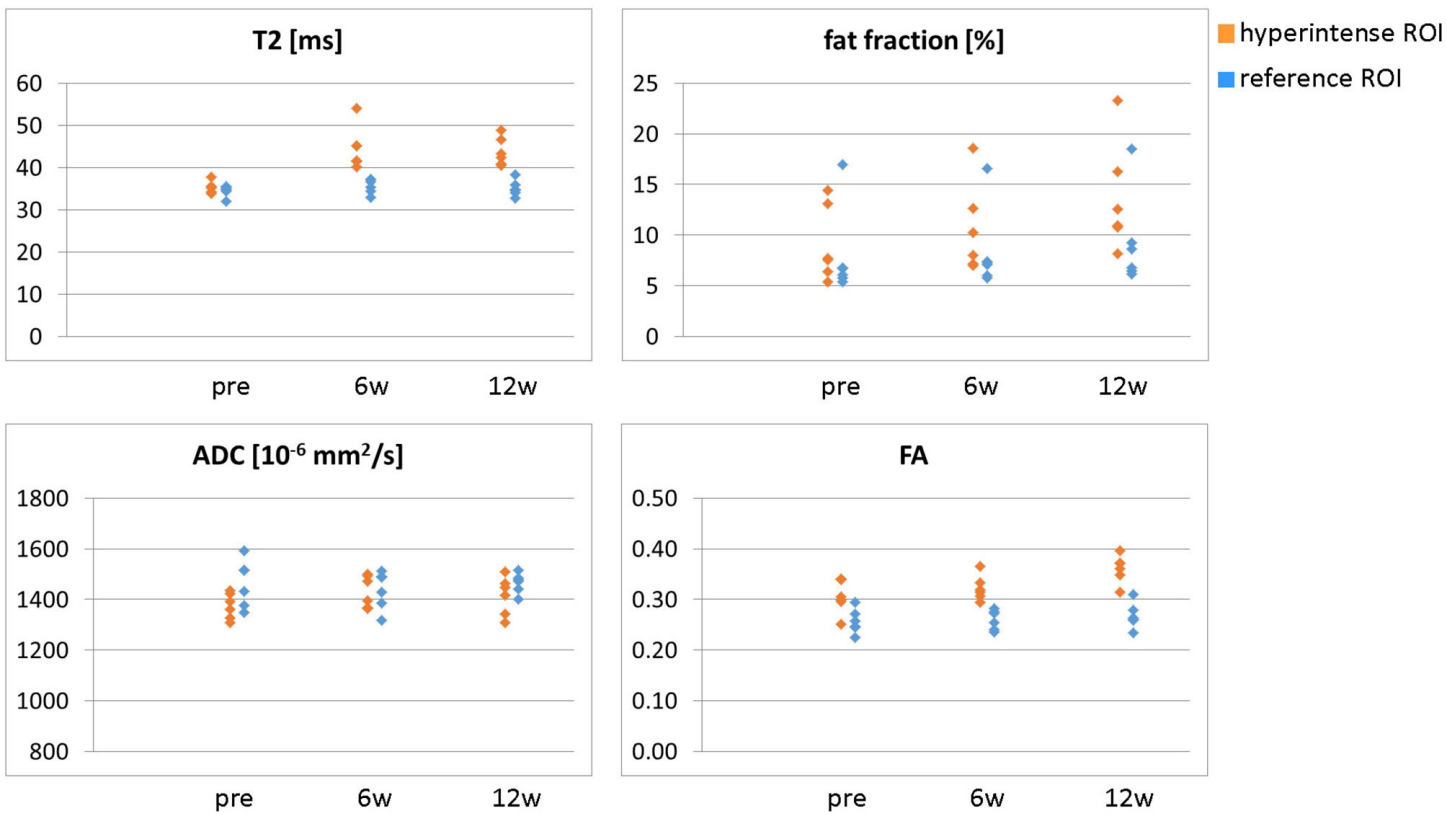
Overview of all ROI mean values (T_2_, fat fraction, ADC, and FA) in the hyperintense (orange) and reference ROIs (blue) for all six patients at time points pre-BTX, 6 weeks (6w), and 12 weeks (12w) post-BTX. ROI, region of interest; BTX, botulinum toxin A; ADC, apparent diffusion coefficient; FA, fractional anisotropy.

Hyperintensity on T_2_w scans and considerably increased T_2_ (by approximately 10 ms compared with baseline) were observed in all patients in the GM and—more pronounced—in the S at the injection sites at 6w and 12w post-injection (Figures 1, 2). T_2_ in the reference region was constant over time, with a group mean value across six patients (±standard deviation STD across six patients) of 35 ± 1, 36 ± 2, and 36 ± 2 ms at time points pre-BTX, 6w, and 12w, respectively (Figure 3, top row, and Figure 4). In the hyperintense ROI, the mean value (±STD) across six patients increased from 35 ± 1 ms pre-BTX to 45 ± 2 ms at 6w and 44 ± 2 ms at 12w post-BTX injection. There was no hyperintensity in the S of patient 6, as expected, since this patient was not treated in the S (Figure 2, right column).

The T_2_ effect, however, was spatially limited and was only observed in parts of the muscles. That was mainly obvious in the S when looking at the extent of the hyperintense ROI (Figure 1, middle row). The hyperintense ROI covered 9–15% of the total GM, GL, and S volumes in all patients except in patient 6, and 43% of the volume of the GM+GL in patient 6 treated the GM and GL only (Table 2).

**Table 2:** Volume of treated muscles and percentage of tissue with elevated T_2_, i.e., relative ROI size at 6 weeks post-BTX.

**Patient number**	**Treated muscles**	**Total volume of treated muscles (cm^3^)**	**ROI size (cm^3^)**	**Percentage of tissue with elevated T_2_ = relative ROI size %**
1	GM, GL, S	225	19.6	9
2	GM, GL, S	223	32.2	14
3	GM, GL, S	231	32.7	14
4	GM, GL, S	166	24.3	15
5	GM, GL, S	203	21.3	10
6	GM, GL	58	25.2	43

*ROI, region of interest; GM, m. gastrocnemius medialis; GL, m. gastrocnemius lateralis; S, m. soleus; BTX, botulinum toxin A*.

ADC did not change in these hyperintense ROIs (Figures 3, 4): group mean values ± STD were 1.37 ± 0.05 ^*^ 10^−3^ mm^2^/s pre-BTX, 1.43 ± 0.09 ^*^ 10^−3^ mm^2^/s at 6w, and 1.41 ± 0.08 ^*^ 10^−3^ mm^2^/s at 12w. Also in the reference ROIs, there was no change in the mean ADC: 1.46 ± 0.09 ^*^ 10^−3^ mm^2^/s pre-BTX, 1.44 ± 0.08 ^*^ 10^−3^ mm^2^/s at 6w, and 1.47 ± 0.04 ^*^ 10^−3^ mm^2^/s at 12w. FA group mean ± STD was 0.30 ± 0.03 pre-BTX and increased to 0.34 ± 0.02 at 6w and 0.36 ± 0.03 at 12w. The FA values in the reference ROIs were stable: 0.26 ± 0.02 pre-BTX, 0.26 ± 0.02 at 6w, and 0.27 ± 0.03 at 12w.

There was a trend of an increase in the fat fraction in the hyperintense ROI at 12w: group mean values ± STD were 9.1 ± 3.7% pre-BTX, 10.6 ± 4.4% at 6w, and 13.7 ± 5.4% at 12w. Patient 2 was overweight (BMI > 85^th^ percentile) and showed higher fat fraction values than the rest of the group, also in the reference ROIs (top data points in Figure 4, top right).

## Discussion

The effect of BTX injections into the calf muscles of pediatric CP patients was quantitatively assessed using MRI over 12 weeks. Our study showed substantial elevation of T_2_ values at the BTX injection sites in pediatric CP patients up to 12w post-treatment. It is not a direct visualization of BTX bolus, as reported by Elwischger et al. (23). In their study in healthy and spastic biceps brachii muscles in adults, the convection of the fluid depot was visualized as hyperintense areas in images at the injection site in a few minutes after BTX injection. The bolus expanded primarily along the muscle fibers and dispersed to a longer distance in healthy compared with spastic muscles. The distribution process was not completed at the second MRI at 11 min after injection, and therefore, it would be of interest to know the effect of BTX on muscle tissue after a more extended period. In our study, the bolus had already dispersed by the time of the MRI exam at 6w after the BTX injections. Our results are in line with O'Dell et al. (24). In their study, 200 units of BTX were injected in various leg muscles of adult patients who suffered a stroke (total dose of 200 units). An increase in T_2_ was observed 2 and 3 months after BTX injection. Schroeder et al. (25) observed a hyperintensity in T_2_w images up to 1 year in the gastrocnemius muscles of two healthy adults injected with a single dose of BTX (75 units). As already pointed out in these two studies, the increase in T_2_ is presumably caused by an increase in the extracellular space (ECS) around the atrophic muscle fibers after treatment. In Schroeder et al.'s study, the treated muscles' biopsies showed increased connective tissue and enlargement in ECS (25).

In the absence of fat-suppression techniques, global T_2_ increases with an increase in fat content, with fat having a longer T_2_ than water. In our study, we acquired the T_2_ maps with fat suppression. However, residual fat signal from an imperfect fat suppression may influence the measured T_2_ values (26). Since the fat fraction did not change at 6w post-BTX, the elevated T_2_ represents a T_2_ increase in muscle water and is presumably caused by an ECS increase.

Regarding the diffusion results, our ADC values of approximately 1.4 ^*^ 10^−3^ mm^2^/s are in the same range as in adult calf muscles (11). The FA values around 0.3 to 0.4 are in line with previously published results in healthy adult volunteers (27). We could not detect an increase in ADC as one would expect with an increased ECS. However, changes in muscle fiber size (i.e., fiber diameter) also have to be taken into account. BTX injections cause a decrease in muscle fiber size and, therefore, atrophy of the muscle (28). In the work by Berry et al. (29), a simulation of a standard single-echo DTI experiment in BTX-treated muscle with an increased ECS and decreased fiber size resulted in minimal changes in ADC, as ADC decreases with fiber size. As shown in the same study (29), FA is more sensitive to fiber size changes than ADC, as it increases with decreasing fiber size. The upper simulation of the BTX-treated muscle with reduced fiber size and increased ECS resulted in an increased FA. In our study, we observed a slight increase in FA over time post-BTX. Our results are also in line with a study in a surgical denervation model in rats causing muscle atrophy: no significant difference in muscle ADC was found between the control group and the denervated group, while FA increased in the atrophic muscle (30). These studies and our observations indicate that an increase in ECS and a simultaneous decrease of muscle fiber size by the application of BTX may counteract the effect on ADC, thus resulting in stable ADC values, whereas FA increases.

Concerning the fat quantification, we only observed a trend for an increase in fat fraction at 12w post-BTX. The fat fraction values had a large standard deviation across the ROIs. Chemical denervation by BTX—like denervation in general—leads to a partial replacement of muscle fibers by fat (3). However, the increase in fat content is only small after BTX treatment. A mild increase in the number of fat cells 12 months after a single dose of BTX was reported in biopsy calf muscle specimens (25). A small amount of lipids in the range of 1% relative to total muscle cross-sectional area (compared with almost 0% in controls) was detected in sections of rat muscle 3 months after a single dose of BTX (28). Such a slight change in fat fraction was challenging to detect in our study. Possibly, the fat fraction may further increase and be better detectable after 12w post-BTX until the BTX effect wears off and recovery of muscle morphology sets in Multani et al. (3).

As expected in our patient cohort, clinical examination showed no significant functional improvement after BTX. In this case, the intention of the injection was not the treatment of the equinus but to test the weakening of the muscle for a risk evaluation of the planned lengthening surgery (13). Therefore, their toe-walking remained unchanged after injection. This is in line with the general observation that a response to equinus treatment with BTX is barely detectable in children over 6 years (3). The patients in this study all showed fixed calf muscle contractures in the clinical examination. BTX thus would only have an effect on the additional functional part of the equinus and on stability. Muscle strength as assessed with MMT did not change in our patient cohort after injection. The decision to proceed with the planned lengthening surgery was based on the post-BTX gait analysis. Since the gait pattern did not deteriorate after BTX injection, all six patients were scheduled for surgery, as poor surgery outcomes due to further muscle weakening were considered unlikely.

T_2_w images and T_2_ maps showed hyperintensity and increased quantitative values, respectively, but only in parts and not in the muscles' complete cross section. Based on animal and human data and clinical experience, BTX diffusion is likely to occur up to a distance of 4.5–5 cm from the injection site (to some extent, even into adjacent muscles). This depends on the dosage and volume injected (31) and on the muscle fiber structure and presence of spasticity (23). The hyperintense region's extent in our study in CP patients with muscle contractures was smaller than this maximum distance. The same review (31) also points out that directly detecting BTX spread in the human muscle is difficult. T_2_-weighted imaging may be a potential tool to assess this spread non-invasively.

Our study has some limitations. The number of patients was small. Therefore, we refrained from using statistical testing for differences in the MRI results and solely used descriptive statistics in our preliminary report. Due to the relatively large standard deviations in the ROIs, a clear interpretation of the fat fraction and diffusion maps was difficult. However, the trends in the time courses after treatment were compelling and in line with the existing literature (e.g., simulations, biopsy data, and animal studies). The hyperintensity on T_2_w images and T_2_ maps up to 12 weeks after BTX treatment was clearly visible and confirmed the findings of the two published pilot studies in adults (24, 25). We performed this pilot study in children over 8 years of age since they could follow instructions in the scanner and lie still without sedation. It would be interesting to perform the experiments in younger children without fixed contractures, who are the main target group for BTX treatment, but then sedation might be necessary to avoid motion artifacts in the MR images. We only tested one specific treatment protocol with dose, dilution, and position of injection sites suited for the preoperative testing but not optimized for equinus treatment. The analysis of more patients within the current study and more studies on this topic are necessary to draw firmer conclusions.

The method may be applied in future studies with other BTX treatment protocols to quantify BTX-induced effects on muscle tissue and to investigate the spatial BTX distribution. A spatially limited extent of the BTX-induced effect, i.e., if the spread of BTX does not fully cover the zone of the motor end plates in the muscle (31), may help to explain a limited treatment effect on the gait and on the clinical scores of CP patients. Based on the MRI results, a revision of the applied treatment protocol regarding dose, dilution, or injections sites may be considered. The recommended total dose was reduced in the last years after serious side effects, especially in severely affected CP patients (2, 15). As discussed in a recent review (31), in relatively large muscles like leg muscles, it may be beneficial to use higher doses or higher dilutions (if a dose increase is not beneficial) and divide the dose over multiple injection sites.

In conclusion, our study shows that MRI is a useful tool for monitoring the spatial distribution of BTX treatment effects. It is especially well-suited to non-invasively determine BTX-induced changes and therapeutic responses in a pediatric population. To our knowledge, our study is the first to employ MRI to monitor BTX treatment effects in children. Using the proposed method as a monitoring tool in future studies will provide more detailed insight into the therapeutic effect of BTX in muscles of CP patients and may influence the treatment process (e.g., injection protocol).

## Data Availability Statement

The datasets presented in this article are not readily available because restrictions apply to DICOM data originating from the MRI scanner. Requests to access the datasets should be directed to CW, claudia.weidensteiner@unibas.ch.

## Ethics Statement

The studies involving human participants were reviewed and approved by Ethikkommission Nordwest- und Zentralschweiz, Basel, Switzerland. Written informed consent to participate in this study was provided by the participants' legal guardian/next of kin.

## Author Contributions

RB, ER, CW, OB, and MG designed the study. RB supervised the project. CW, PM, and TH performed MRI experiments. KB-S, ED, and JR performed and analyzed clinical examinations. CW, TA, and PM analyzed MRI data. XD, FS, and PM developed analysis tools. XD and FS assisted with MRI experiments. CW wrote the manuscript. RB, OB, XD, FS, ED, JR, PM, TA, and MG revised and edited the manuscript. The final manuscript version was approved by all authors.

## Conflict of Interest

The authors declare that the research was conducted in the absence of any commercial or financial relationships that could be construed as a potential conflict of interest.
